# Molecular modeling, docking and dynamics analysis of lipid droplet associated enzyme Ypr147cp from Saccharomyces cerevisiae

**DOI:** 10.6026/97320630017132

**Published:** 2021-01-31

**Authors:** Kishore Sesham, Naresh Kumar Manda

**Affiliations:** 1 Department of Anatomy, All India Institute of Medical Sciences (AIIMS), New Delhi, India; 2 Department of Biochemistry, School of Life Sciences, University of Hyderabad, Hyderabad, Telangana-500046, India;

**Keywords:** Molecular modeling, docking, GXSXG motif, Alpha Beta Hydrolase Domain (ABHD), Lipid Droplet (LD), Triacylglycerol (TAG)

## Abstract

Ypr147cp of Saccharomyces cerevisiae was localized to lipid droplets. The recombinant Ypr147cp showed both triacylglycerol lipase and ester hydrolase activities. Knock out of YPR147C led to accumulation of TAG in ypr147cΔ when compared to wild type (WT). Transmission electron microscopic analysis of ypr147cΔ cells show increased lipid bodies. Moreover, the lipid profiling confirmed the accumulation of fatty acids derived from neutral and phospholipids in ypr147cΔ cells. Sequence analysis of Ypr147cp show the presence of an a/b hydrolase domain with the conserved GXSXG lipase motif. The YPR147c homology model was built and the modeled protein was analysed using RMSD and root mean square fluctuation (RMSF) for a 100 ns simulation trajectory. Docking the acetate, butyrate and palmitate ligands with the model confirmed covalent binding of ligands with the Ser207 of the GXSXG motif. Thus, Ypr147cp is a lipid droplet associated triacylglycerol lipase having short chain ester hydrolyzing capacity.

## Background

Saccharomyces cerevisiae's Ypr147cp, previously known as bifunctional enzyme which acts as triacylglycerol lipase and short chain ester hydrolase, null mutant results in the accumulation of both triacylglycerol and fatty acids derived from neutral lipids and phospholipids as well as an increase in the quantity of lipid droplets, contains an alpha/beta hydrolase domain with a conserved GXSXG lipase motif [1] localizes to lipid droplets [2]. GFP-fusion protein localizes to the cytoplasm [3] and is induced in response to the DNA-damaging agent MMS [4]. Its role in the lipid metabolism plays a significant role in lipid homeostasis. However, activity of lipase has not been studied in any detail and there are no bioinformatics data to confirm the functionality, evolutionary relationship, substrate specificity and the role of this protein in lipid breakdown. In the present study, we report the molecular modeling, docking and dynamics analysis of Saccharomyces cerevisiae lipid droplet associated enzyme Ypr147cp to confirm its activity as triacylglycerol (TAG) lipase and short chain ester hydrolase.

## Materials and Methods:

### Homology modelling of YPR147C:

The YPR147C sequence was retrieved from UNIPROT (Universal Protein Resource), and the template for homology modeling were searched from the Blast [5] against protein databank [6]. There were no close similar structures in the pdb, thus templates were searched in swissmodel database [7], two templates with pdb id: 26A5 and 2ZSH showed positive identities. ClustalW [8], provided the percentage similarity between YPR147C and 2A65, 2ZSH are 28% and 20.9% . Based on the swissmodel database templates and the ClustalW alignment score the structure for YPR147C was modelled using multitemplate and loop refinement modeling using MODELLER9v7 [9,10]. Modelled structures were analyzed for their DOPE score [11], and finally high score model was viewed through PYMOL [12], later validated through PROCHECK and Zscore [13,14] analysis.

### Molecular Docking:

Molecular docking is important computational procedure performed to find out the exact binding site and most favorable interacting regions between small molecules and protein, which fit geometrically and energetically by using Auto Dock 4.2.3 software [15]. Three-dimensional structure of small molecules was built from 2D structure and using Discovery studio 3.5 software optimized geometry. The modelled YPR147C was optimized and used as input for AutoDock tools. Water molecules were added by default, and polar hydrogen were added using the MGL tools interface [16]. Pnp acetate, pnp butyrate, pnp deconate, pnp dodeconate, pnp octonate, pnp oleate, pnp palmitate and pnp stereate binding sites and type of interactions were performed using the Lamarckian genetic algorithm implemented in AutoDock 4.2.3. For each docking simulation 50 conformers were generated and analyzed for least binding free energy. Docking results were compared using X-score v1.2.1 [17], a consensus scoring function where it calculates the negative logarithm of dissociation constant of ligand to protein, and predicts the relative binding energy (Kcal/mol) of the ligand.

## Results and Discussion:

**Figure 1:**
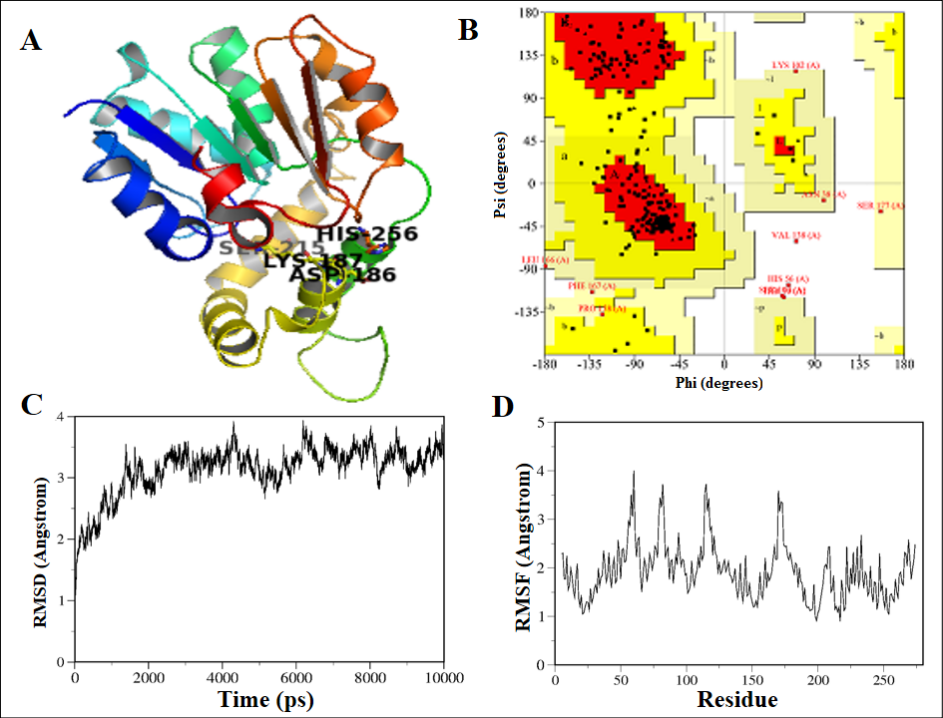
Homology modelling and MDS (A) The predicted 3D model of YPR147C after clustering; (B) Ramachandran plot analysis of the built model; (C) RMSD graph of the model obtained after the 100 ns simulation run; (D) RMSF of the amino acids plotted using the trajectories obtained by the 100 ns MDS.

**Figure 2:**
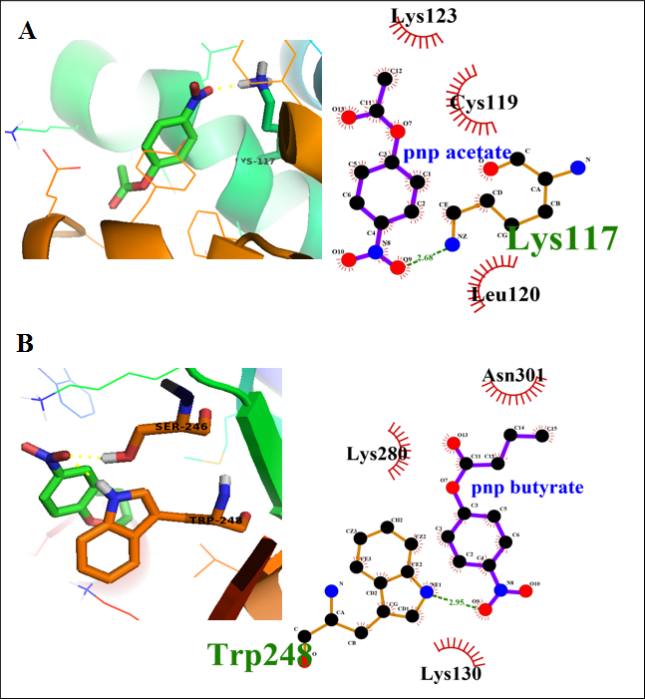
YPR147C docking interactions with pNP substrates Covalent bond formation between the YPR147C model with (A) pNPA; (B) pNPB highlighting the interactions with specific amino acids.

**Figure 3:**
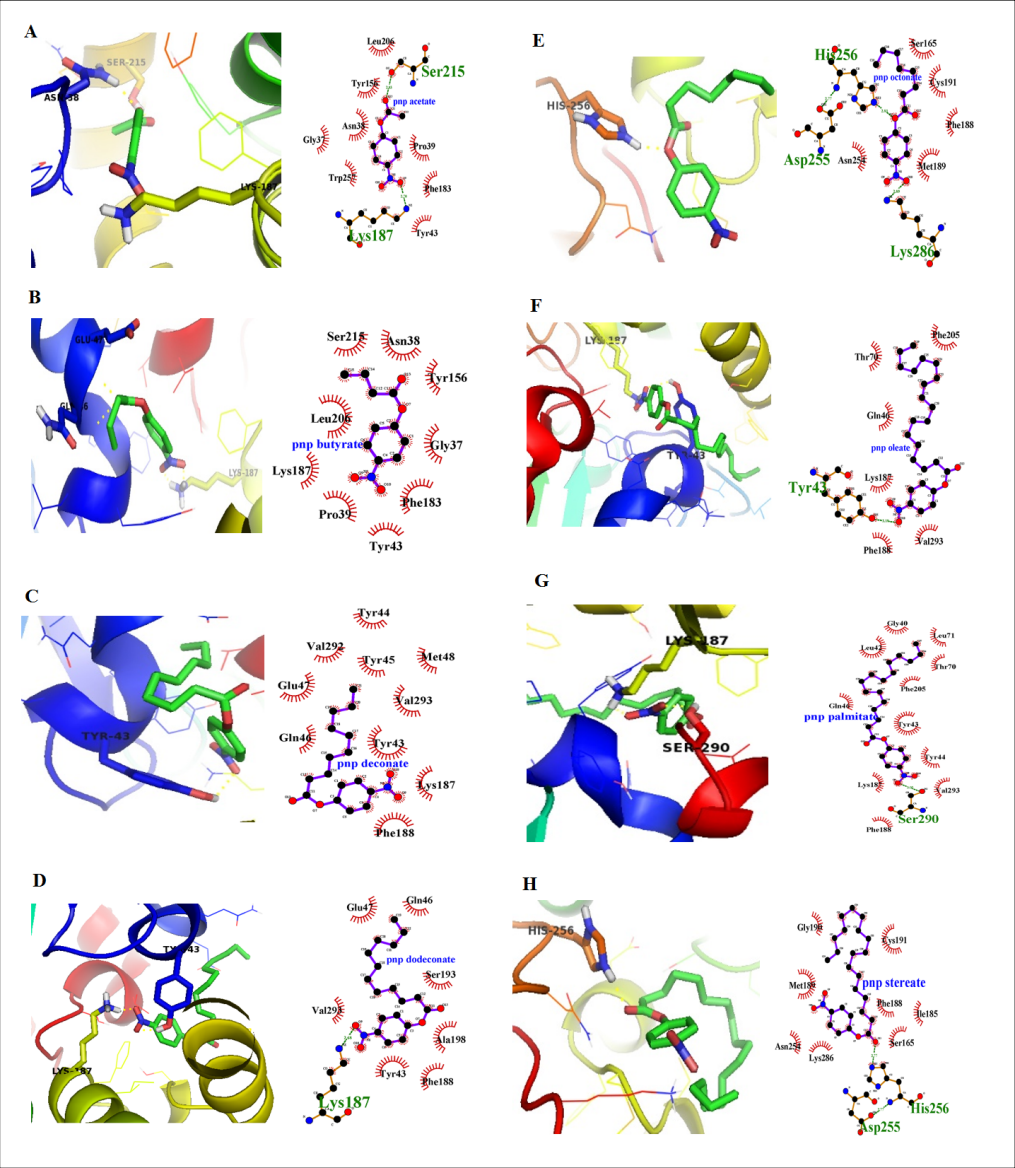
YPR147C Docking Interactions with pNP substrates Covalent bond formation between the YPR147C model with (A) pnp acetate (B) pnp butyrate (C) pnp deconate (D) pnp dodeconate (E) pnp octonate (F) pnp oleate (G) pnp palmitate (H) pnp stereate highlighting the interactions with specific amino acids with YPR147C.

Homolgy model and Molecular docking studies:

The sequence of YPR147C was retrieved from Uniprot and its corresponding sequence id is Q05622, contains 304 amino acids. The important step in homology modeling is to select an appropriate template structure for constructing the target model. This sequence was subjected to similarity search against Protein Data Bank using the Blast tool, unfortunately no hits found in the PDB. Swiss model database provided structural hits and its alignment pattern against the query sequence. The selected templates were a chain of 2A65 and 2ZSH. Using ClustalW the sequences of templates and query sequence were aligned to understand the conserved residues and gap inserts, the percentage similarity in between YPR147C and templates 2A65 and 2ZSH found to be 38% and 20.9%. The resulting alignment file was used as input for Modeller to generate 3D models using the advanced modeling tutorial package in MODELLER 9v7. Since the templates do not found the last 30 residues, corresponding residues from 274-304 were not modeled. The initial 3D models of YPR147C were energy minimized to release the bad atomic contact and unreasonable local structural conformations. Final model with Dope score -70340.663 was selected for further validation. Validation of a 3D model is an essential step to check the stereo chemical parameters and accuracy of the overall packing. The Z-score indicates the overall model quality and is used to check whether the input structure is within the range of scores and the Z-score of the template and query model was -4.97 (Figure 1A). The assessment of main-chain and side-chain residues for selected model was performed using Procheck-Ramchandran plot analysis. The plot showed 82.6% of the residues in the core region i.e. favorable region, 13.8 % in the allowed region and 1.6 % in disallowed region (Figure 1B). Based on the RMSD (Figure 1C), RMSF (Figure 1D) and other results the final model proved to be good enough to be a starting point for further docking studies. The 3D structure of YPR147C is displayed in (Figure 1). Molecular docking gives the detailed picture of the binding site of selected molecules, its position, and orientations of the protein. This information is crucial as it explains the relationship between molecular properties of complexes. As we already known from the literature that lipases have same catalytic triad as ABHD domain composing Ser-Asp-His. The molecules pnp acetate, pnp butyrate, pnp deconate, pnp dodeconate, pnp octonate, pnp oleate, pnp palmitate and pnp stereate showed binding conformations near the catalytic triad with high binding affinity through formation of hydrogen bonds in the range of 1.7-2.5 Å, the list of binding energies and hydrogen bonds are depicted in (Table 1 and 2 - see PDF). Docking Interactions of pnp acetate (Figure 2A), pnp butyrate (Figure 2B) were obtained. Pnp acetate (Figure 3A) and pnp butyrate (Figure 3B) showed hydrogen bonds with Ser215, Lys187 and Asn38 with binding energy -7.2and -7.0 Kcal/mol, whereas the pnp deconate (Figure 3C), pnp dodeconate (Figure 3D), pnp oleate (Figure 3E) and pnp palmitate (Figure 3F) showed interaction with Lys187 with lesser binding energy -5.7, -4.5, -4.72 and -4.76 Kcal/mol. This was due to steric hindrance caused by amino acid side chains near and around the cavity and long fatty acid chain of molecules. Even lesser binding energy -4.0 and -3.9 Kcal/mol with hydrogen bonding interaction with His256 was observed with pnp octonate (Figure 3G) and pnp stereate (Figure 3H), as the binding site could not accommodate the large molecules.

## Conclusion:

We report the Ypr147cp homology model with root mean square fluctuation (RMSF) over the 100 ns simulation trajectory. Docking the acetate, butyrate and other ligands with the model confirmed covalent binding of ligands with the Ser215 of the GXSXG motif. The model was validated with a mutant Ypr147c with alanine for Ser215 showing no interaction between selected ligands and the mutant protein active site.
